# Special Issue: “Molecular Genetics and Plant Breeding, 5th Edition”

**DOI:** 10.3390/ijms26178439

**Published:** 2025-08-30

**Authors:** Hai Du

**Affiliations:** 1 College of Agronomy and Biotechnology, Chongqing Engineering Research Center for Rapeseed, Southwest University, Chongqing 400715, China; haidu81@126.com or dh20130904@swu.edu.cn; 2 Integrative Science Center of Germplasm Creation in Western China (Chongqing) Science City, College of Agronomy and Biotechnology, Southwest University, Chongqing 400715, China

Complex traits in plants are typically governed by multiple genes, making their genetic analysis a long-standing challenge in plant research [1,2,3,4]. The aim of molecular genetics is to decode the molecular mechanisms underlying heredity and genetic variation [5,6], which control plant traits, thereby establishing a foundational knowledge base for agricultural research. Meanwhile, plant breeding translates these discoveries into practical applications by improving plant traits and developing high-quality crop cultivars [7,8,9]. With the rapid advancement of bioinformatics and multi-omics technologies, integrating large-scale datasets, including functional genomics, transcriptomics, epigenomics, proteomics, metabolomics, and other high-throughput approaches, has emerged as a key research focus [10,11,12,13,14]. This integration accelerates plant research by bridging the genotype-to-phenotype gap and enabling data-driven crop breeding [15,16,17,18,19]. In 2019, the *International Journal of Molecular Sciences* (*IJMS*) launched its first Special Issue on “Molecular Genetics and Plant Breeding,” inviting original research and review articles showcasing advancements in plant molecular genetics, multi-omics, and gene resources across all levels. The first four editions of this Special Issue featured 89 published papers [20,21].

In this fifth edition, we received 12 submissions addressing this topic. After rigorous peer review, five original research articles were selected for publication. Among these, three studies focus on investigating the genetic basis, genetic variation, molecular markers, and functional genes across diverse plant germplasms and population materials (Table 1, contributions 1–3). Medina et al. evaluated 424 alfalfa breeding families under deficit irrigation, identifying 131 significant markers linked to biomass yield via GWAS (Genome-Wide Association Study), including 80 markers that were drought-specific. The authors finally provide an optimized genomic prediction framework to accelerate breeding for drought-resistant alfalfa with maintained biomass yield. Zhang et al. discovered novel genetic loci and candidate genes controlling yield-related traits in soybean (*Glycine max*) using a recombinant inbred line (RIL) population and expression analysis, offering molecular targets for high-yield breeding. Jiang et al. integrated BSA-seq and transcriptomics to unravel key genes and molecular mechanisms for cold resistance in rapeseed (*Brassica napus*), using an F2 population derived from cold-sensitive (M98) and cold-tolerant (D1) lines, providing a foundation for molecular breeding of cold-tolerant varieties.

This Special Issue also covers the evolution, molecular characterization, and genome editing of plant gene resources (Table 1, contributions 4–5). Qian et al. conducted a genome-wide analysis of the potassium utilization-related genes in plants, identifying 376 candidate genes in the *B. napus* ZS11 genome through homology-based retrieval. Their comprehensive bioinformatics approach included global gene identification, chromosomal distribution, microsynteny analysis, *cis*-element profiling, expression analyses (RNA-seq), and transcriptomic validation (RT-qPCR). Functional validation via complementation assays in *Arabidopsis thaliana* confirmed the role of *BnaZSHAK5.2* in root adaptation to low-K^+^ conditions. These findings provide molecular targets for breeding rapeseed with improved potassium use efficiency, particularly for nutrient-deficient soils. Li et al. revealed that *OsPPR674* is essential for mitochondrial RNA editing in rice (*Oryza sativa*), influencing plant growth and reproduction, stress response mechanisms, etc. The findings highlight the critical role of PPR proteins in organelle function, and the study proposes RNA editing regulation as a potential tool for enhancing crop resilience.

The advent of next-generation sequencing (NGS) technologies has revolutionized plant genomics research, enabling unprecedented generation of large-scale sequence data and comprehensive multi-omics datasets across diverse plant species [22,23]. In this post-genomic era, integrative multi-omics analysis has become a cornerstone for genome dissection and functional gene discovery, particularly for uncovering genetic resources governing important agronomic traits in crops [24,25,26,27,28]. The studies featured in this Special Issue exemplify the power of multi-omics approaches in crop improvement, successfully identifying key genes and biomarkers linked to important agricultural traits. These findings not only advance fundamental understanding but also deliver practical genetic resources for plant precision breeding. In the future, a major challenge in plant genetics and breeding will be the effective integration of massive multi-omics datasets with advanced artificial intelligence (AI) methodologies, including machine learning and deep learning algorithms to decode complex biological networks and gene resources [29,30,31,32,33]. Such integration promises to unravel the genetic and molecular basis of plant complex traits, accelerate the development of next-generation breeding strategies, and design optimized models for plant breeding. This synergy between omics technologies and computational analytics in plant molecular genetics and breeding will be critical for addressing global food security challenges in the coming decades.

## Figures and Tables

**Table 1:** Compilation of the 5 contributions in this Special Issue.

Contributions	Species	Purpose	Approaches
1	*Medicago sativa* L.	Molecular markers linked to biomass yield	Genetics and genomics
2	*Glycine max* (L.) Merr.	Quantitative trait loci for node, pod or seed traits	Genetics and molecular biology
3	*Brassica napus* L.	Genes associated with cold stress	Genetics and transcriptomics
4	*Brassica napus* L.	Potassium utilization genes	Bioinformatics, transcriptomics and molecular biology
5	*Oryza sativa*	Gene regulates plant growth and drought	Bioinformatics and molecular biology

## Data Availability

For original datasets, please refer to the published articles within the Special Issue “Molecular Genetics and Plant Breeding, 5th Edition” (https://www.mdpi.com/journal/ijms/special_issues/I7DTV8Q7TN) (accessed on 28 February 2025).
